# Where do the antibiotic resistance genes come from? A modulated analysis of sources and loads of resistances in Lake Maggiore

**DOI:** 10.1093/femsec/fiae025

**Published:** 2024-02-22

**Authors:** Andrea Di Cesare, Stefano Mammola, Raffaella Sabatino, Diego Fontaneto, Ester M Eckert, Michela Rogora, Tiziana Tonsi, Gianluca Corno

**Affiliations:** National Research Council of Italy – Water Research Institute (CNR-IRSA), I-28922 Verbania, Italy; National Research Council of Italy – Water Research Institute (CNR-IRSA), I-28922 Verbania, Italy; Finnish Museum of Natural History (LUOMUS), University of Helsinki, FI-00014 Helsinki, Finland; National Research Council of Italy – Water Research Institute (CNR-IRSA), I-28922 Verbania, Italy; National Research Council of Italy – Water Research Institute (CNR-IRSA), I-28922 Verbania, Italy; National Research Council of Italy – Water Research Institute (CNR-IRSA), I-28922 Verbania, Italy; National Research Council of Italy – Water Research Institute (CNR-IRSA), I-28922 Verbania, Italy; National Research Council of Italy – Water Research Institute (CNR-IRSA), I-28922 Verbania, Italy; National Research Council of Italy – Water Research Institute (CNR-IRSA), I-28922 Verbania, Italy

**Keywords:** anthropogenic pollution, antibiotic resistance genes, antimicrobial resistance, rivers and lakes

## Abstract

Antibiotic resistance genes (ARGs) are abundant in aquatic ecosystems affected by human activities. Understanding the fate of ARGs across different ecosystems is essential because of the significant role aquatic environments play in the cycle of antibiotic resistance. We quantified selected ARGs in Lake Maggiore, its main tributaries, and the effluent of the main wastewater treatment plant (WWTP) discharging directly into the lake. We linked their dynamics to the different anthropogenic impacts in each tributary's watershed. The dynamics of *tet*A in the lake were influenced by those of the rivers and the WWTP effluent, and by the concentration of N-NH_4_, related to anthropogenic pollution, while *sul*2 abundance in the lake was not influenced by any water inflow. The dynamics of the different ARGs varied across the different rivers. Rivers with watersheds characterized by high population density, touristic activities, and secondary industries released more ARGs, while *erm*B correlated with higher numbers of primary industries. This study suggests a limited contribution of treated wastewater in the spread of ARGs, indicating as prevalent origin other sources of pollution, calling for a reconsideration on what are considered the major sources of ARGs into the environment.

## Teaser

Treated wastewaters can have a limited contribution to the spread of antimicrobial resistance genes in anthropogeneically impacted aquatic ecosystems.

## Introduction

Antibiotic resistance genes (ARGs) are widespread in aquatic environments, and their abundance and richness are larger when anthropogenic pollution is high (Zhu et al. 2017). The role the environment plays in the spread and in the transmission of antibiotic-resistant pathogenic bacteria and of ARGs has been widely documented (Larsson et al. 2018), but a number of questions remain open about their dynamics and the factors underlying their ecological success in open waters. Among the different aquatic ecosystems, large lakes in areas exposed to anthropogenic pressure are an important natural reservoir of ARGs (Eckert et al. 2018, Nnadozie and Odume 2019). Resistances reach lakes mainly from their watershed, brought from the tributary rivers, which are collecting ARGs from diffuse (water runoff from agricultural and industrial areas, roads, cities and leisure areas) and/or punctual sources [effluent of wastewater treatment plants (WWTPs), sewage bypasses, untreated discharges] (Pruden et al. 2012, Marti et al. 2014).

Nowadays, the general consensus is that WWTP effluents are identified as a major source of ARGs into surface waters (Rizzo et al. 2013, Czekalski et al. 2014), and a large body of research unveils the impact of WWTP effluents on the microbial communities of the receiving environments, the fate of the ARGs within the natural communities, and the removal efficiencies of WWTPs applying different technologies (Czekalski et al. 2014, 2016, Corno et al. 2019, Sivalingam et al. 2023). The impact of diffuse ARG sources is also well documented, but the interactions between structured environments (complex anthropogenically impacted areas, rivers, lakes) coupled with the impact of climatic and meteorological parameters, reduce our understanding of the impact caused by these sources on the spread of the ARGs (Di Cesare et al. 2017, Yu et al. 2018).

In this study, we investigated the dynamics of five ARGs in the watershed of Lake Maggiore, a deep oligotrophic subalpine lake shared between Italy and Switzerland (lake surface: 212 km^2^, max depth: 370 m). This lake is one of the most studied lakes in the world and it receives waters from one of the largest catchment areas (6659 km^2^) in the Alpine Region, shared almost equally between Italy and Switzerland. This area hosts about 640000 inhabitants (CIPAIS 2003) and a large number of seasonal tourists, mainly in spring and summer (the number of tourists hosted in the watershed of the Lake in 2015–6 was between 4 and 5 million; USTAT Canton Ticino 2017, ISTAT 2017a). From 2013, the horizontal and vertical distribution of ARGs in Lake Maggiore has been assessed monthly by monitoring programs funded by the International Commission for the Protection of Italian-Swiss Waters (Di Cesare et al. 2015, CIPAIS 2018, Eckert et al. 2019a, Di Cesare et al. 2020, CIPAIS 2021). However, the relative contribution of the ARGs released from its watershed through the tributary rivers or from the WWTP effluents directly discharged into the lake to the resistome of the lake has never been investigated. We sampled the lake, its six main tributaries (i.e. Bardello, Strona, San Bernardino, Ticino, Toce, and Tresa), and the effluent of the WWTP located in Verbania, one of the largest (and the few) WWTPs discharging directly in the lake, over the course of one year: overall we analyzed samples covering ~80% of the annual water inflow of Lake Maggiore. The Strona and Toce rivers, gathering waters from watersheds with different human use, were sampled shortly before the former flows into the latter, at a short distance from the Toce estuary into Lake Maggiore. For all sampling sites, we quantified five ARGs (present on the bacterial chromosome or within plasmids) by means of quantitative real time PCR (qPCR). The selection of ARGs was guided by the results obtained by the CIPAIS long-term monitoring of Lake Maggiore, focusing on genes associated with resistance to the most commonly used antibiotics in the Lake Maggiore area (CIPAIS 2018). Two genes, *sul*2 and *tet*A (encoding for the resistance to sulphonamides and tetracyclines, respectively) were almost always present in the lake (Di Cesare et al. 2015); *bla*_CTX-M_ (encoding for resistance to β-lactams) followed a seasonal dynamic within the lake bacterial community (Eckert et al. 2019a); *erm*B and *qnr*S (encoding for the resistance to macrolide-lincosamide-streptogramin and quinolones, respectively) were sporadically detected in the lake, and are classified as high-risk ARGs for human health (Zhang et al. 2021). We also measured biotic and abiotic factors that may influence the bacterial community and their associated ARGs: bacterial cell number and size distribution, water flow, nitrogen and phosphorus compounds and total organic carbon. We then analyzed results taking into consideration the level and the type of anthropogenic pressures (urban, agricultural, industrial, touristic) impacting in each tributary's watershed. For this reason, we collected specific data (number and density) on resident population, primary and secondary industries, hotels, as well as the number and size of WWTPs in the basin of the lake. Finally, using the WWTP of Verbania as a model, we approximated the impact of treated effluents in the different watersheds and directly into the lake.

## Methods

### Site sampling, water collection and processing

**Figure 1.**
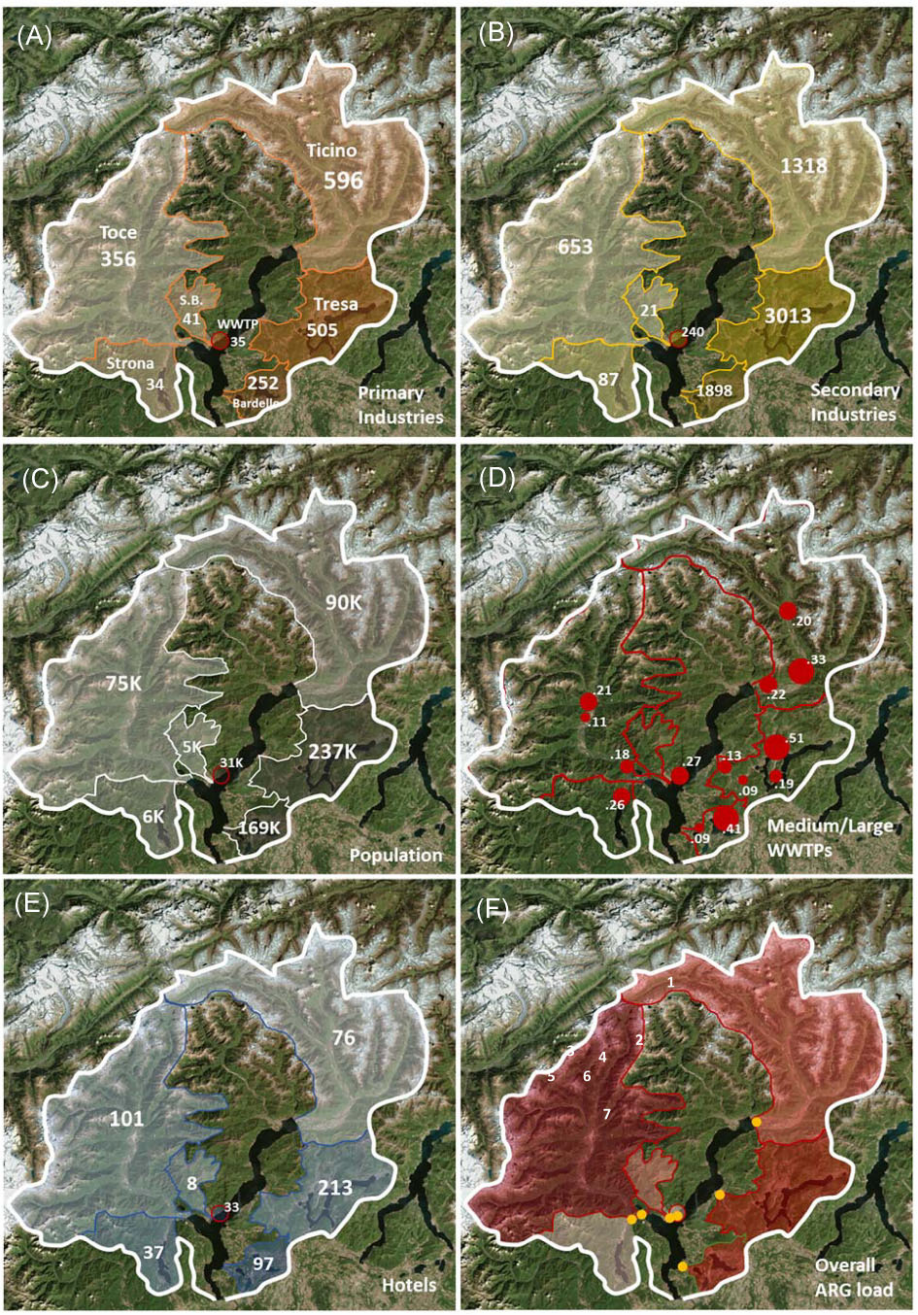
Anthropogenic pressures in the different watersheds in Lake Maggiore basin: (A) primary industries (agriculture, mining), (B) secondary industries (factories, transformation industries), (C) resident population, (D) large and medium-sized WWTPs, (E) hotels, recreational structures, (F) overall ARGs load from the watershed into the lake per year. The name of the respective rivers in (A) refers to all panels. The orange dots in (F) represent the sampling points for each watershed (1: Ticino, 2: Tresa, 3: Toce, 4: WWTP effluent, 5: San Bernardino, 6: Strona, 7: Bardello). The red circle in (A), (B), (C), (E) and (F) represents the effluent of the WWPT of Verbania and its respective contributions. The numbers refer to the absolute value for each parameter in the respective watershed, while the color refers to the density of each parameter in each watershed (bright: lower, dark: higher). In (D), red circles are proportional to the size of the WWTP and numbers represent the water flow of each WWTP effluent (in m^3^ sec^−1^).

We collected composite (0–20 m) water samples (>5 L) from Lake Maggiore at the CIPIAS sampling site of Ghiffa [WGS84 latitude (N) 45.951666, longitude (E) 8.635106], at the mouth of the rivers Bardello [latitude (N) 45.839086, longitude (E) 8.623243, discharge of 9.15 × 10^7^ m^3^/year], San Bernardino [latitude (N) 45.932218, longitude (E) 8.570869, discharge of 2.24 × 10^8^ m^3^/year], Ticino [latitude (N) 46.158303, longitude (E) 8.888943, discharge of 2.16 × 10^9^ m^3^/year] and Tresa [latitude (N) 45.995001, longitude (E) 8.728796, discharge of 7.41 × 10^8^ m^3^/year], at River Toce just before the junction with River Strona [latitude (N) 45.936036, longitude (E) 8.444067, discharge of 2.11 × 10^9^ m^3^/year], about 3 km from the mouth, at River Strona, before its confluence with the River Toce [latitude (N) 45.875565, longitude (E) 8.409175, discharge of 1.32 × 10^8^ m^3^/year)] and from the effluent of the WWTP of Verbania [latitude (N) 45.928393, longitude (E) 8.573602, discharge of 8.51 × 10^6^ m^3^/year] (Fig. 1F) (Di Cesare et al. 2016a, CIPAIS 2018). We sampled the sites every two months from July 2015 to May 2016. An aliquot of water was prefiltered on a 126-µm mesh size net and 1.5 ml of filtered water was fixed with formalin (1% final concentration) to be analyzed by flow cytometry. An aliquot (1 L) was processed to measure the main chemical variables. Another aliquot was prefiltered on a 10-µm mesh size net to remove large particles and metazoan and a known volume of filtered water (between 100 ml and 1 L) was further filtered onto 0.22-µm polycarbonate filters and stored at –20°C for the molecular analyses.

Each river's discharge and the corresponding watershed area were obtained from the CIPAIS Annual Report 2017 (CIPAIS 2018); population, number of hotels, number of primary (i.e. producing raw materials like farms and caves) and of secondary industries (i.e. manufacturers) in each watershed were obtained from the Annual Reports of the Italian Institute of Statistics (ISTAT 2017a,b) for the Italian part of the lake basin, and from the Report by the Dipartimento del Territorio del Canton Ticino (2018) for the Swiss part of the basin (Table S1).

### Chemical variables and bacterial abundance measurements

We measured total organic carbon (TOC), total nitrogen (TN), nitrate (N-NO_3_), ammonium (N-NH_4_), total phosphorus (TP) and reactive phosphorus (RP) as previously described (Sathicq et al. 2022) and according to standard methods for freshwater samples (APHA/AWWA/WEF 2012). We analyzed the bacterial abundance and size distribution by flow cytometry (BD Accuri C6) after staining of the water samples with SYBR Green I (final concentration 1%, Life Technologies). We quantified single bacterial cells, microcolonies (up to 10 cells) and bacterial aggregates (>10 aggregated cells); the limits of the counts and the gates in the cytograms were determined as previously described (Corno et al. 2013). The identification of these three gates allows the comparison of microbial communities where the free-living life style is prominent from those where aggregates and particle-attached bacteria are more abundant. An increase in aggregational forms, when supported by other chemical and physical data, suggests environment more disturbed, either by ecological (Callieri et al. 2016) or anthropogenic (Eckert et al. 2019b) stressors.

### DNA extraction and 16S rRNA gene and ARG quantification

Two quarters of each filter (see section Site sampling, water collection and processing) were processed for DNA extraction using the commercial kit DNeasy UltraClean (Qiagen), following the manufacturer's instructions. Each DNA sample was 2-fold diluted and analyzed to quantify the genes 16S rRNA, *sul*2, *tet*A, *bla*_CTX-M_, *erm*B and *qnr*S by qPCR using primers, protocols and programs as previously published (Di Cesare et al. 2015). The potential PCR inhibition was tested by the dilution method as previously described (Di Cesare et al. 2013) and no inhibition was determined. The efficiency of reaction, R^2^ and the limits of quantification were measured as previously described (Bustin et al. 2009). The average value ± standard deviation of the efficiency of reaction and R^2^ considering all the reactions performed were 95.56 ± 16% and 0.98 ± 0.01, respectively. The limits of quantification (LOQ) were 57, 41, 11, 73, 54 and 62 copy/µL for the 16S rRNA, *sul*2, *tet*A, *bla*_CTX-M_, *erm*B and *qnr*S genes, respectively. The gene abundance was expressed converting ng/reaction in gene copy/reaction as previously shown (Di Cesare et al. 2013). The ARG abundances were expressed in relative manner dividing the copy number of ARG per the copy number of the 16S rRNA gene. In cases where the value of the abundance of each gene was below the LOQ but higher than three copy/reaction (the theoretical limit of qPCR, Bustin et al. 2009), the gene was considered present but not quantifiable (NQ). The treating of the values of the gene abundances between the two replicates per sample was done as previously described (Di Cesare et al. 2015), taking into account the limits of having two pseudo-replicates per sample.

### Statistical analysis

We ran all analyses in R version 4.1.0 (R Core Development Team 2021), using the "tidyverse" suite version 1.3.1 (Wickham et al. 2019) for data handling and visualizations. R code to reproduce the analyses is available in GitHub (https://github.com/CNR-IRSA-MEG/metaR/tree/master/lib/CIPAIS%20monitoring%202015-2016).

First, we used Pearson's *r* correlations to explore, across sampling dates (n = 6), the correlation between the abundance of the genes in the tributary rivers and in Lake Maggiore.

Next, we ran two sets of generalized linear mixed models to explore the effect on the presence/absence (first set of models) or abundance (second set of models) of the five genes as response variable of the biotic and abiotic variables of the water in each sampling site/date (total cell number, number of aggregates, number of microcolonies, N-NO_3_, N-NH_4_, TN, TOC, RP and TP) as predictors. We constructed regression models across all sites and sampling dates, with a total sample size of 52 observations. In regression analyses, we followed the general protocol from Zuur and Ieno (2016).

Prior to model construction, we visually inspected variable distribution, presence of outliers, multicollinearity among predictors (using pairwise Pearson's *r* correlations) and balance of factor levels (Zuur et al. 2010). As a result of multicollinearity analysis, we kept as continuous independent variables total cell number, TN, N-NH_4_, TP and sampling date (all having pairwise | r | proxy of load of <0.7), while we excluded the number of aggregates, number of microcolonies, N-NO_3_, TOC and RP) (Fig. S1). Also, we scaled to a mean of zero and a standard deviation of one all continuous independent variables to obtain comparable effect sizes and facilitate convergence of regression models.

We fitted models using the R package "glmmTMB" version 1.1.1(Brooks et al. 2017). For the presence/absence models, we assumed a Bernoulli distribution and a complementary log–log link function (clog-log), as is recommended for unbalanced distributions between zeros and ones (Zuur et al. 2009). For the concentration models, we assumed a Gaussian distribution. The structure of the models, in R notation, was:


\begin{eqnarray*}
{\rm y} \sim {\rm sampling\,\, date} &+& {\rm total\,\, cell\,\, number} \\
&& +\quad {\rm TN} + {\rm N} - {{\rm NH}_4} + {\rm TP} + (1|{\rm Site})
\end{eqnarray*}

where y (dependent variable) is the presence/absence or abundance of the five genes. Note that, because of the high prevalence of zeros, we could not run the abundance model for the *bla*_CTX-M_ gene, resulting in a total of nine individual models. In all models, we included sampling site as a random intercept structure (factor with eight levels) to account for the non-independence of samples (i.e. pseudo-replication stemming from repeated measures at each site). We carried out model validation by inspecting model residuals with the *check_model* function in the package "performance" version 0.9.0.6 (Lüdecke et al. 2021).

Finally, we explored the role of different geomorphological and anthropogenic factors of each tributary's watershed in driving the abundance of ARGs. For this, we selected eight catchment-level variables: bacterial aggregates, waterflow, catchment area, total population, number of primary industries (farms, mines), number of industries and number of hotels (a proxy for the touristic pressure). Using Pearson's *r* correlation, we explored the correlation between the abundance of the five ARGs genes and these eight variables. We also used principal component analysis (PCA) to reduce the dimensionality of this dataset and identify the underlying patterns in the relationships among variables. First, we standardized data to ensure that each variable had equal weight in the analysis. We used standardized variables to calculate the covariance matrix, which describes the relationships between the variables; next, we computed the eigenvectors and eigenvalues of the covariance matrix and used the eigenvectors to transform the data into principal components.

## Results

### Chemical parameters and bacterial abundance and size distribution

Apart for TOC and N-NH_4_ that, as average values, were present in higher concentrations in River Bardello (3.28 and 0.112 mg/L, respectively), all the other measured chemical variables, that is, TN, N-NO_3_, TP and RP had higher concentrations in the effluent of the WWTP (6.33, 5.80, 0.536 and 0.468 mg/L, respectively) (Table S2). River Bardello also showed the second highest concentration of TN, TP, N-NO_3_ and RP. Bacterial cell numbers were higher in River Tresa, both as an average value (4.28 × 10^6^ cell/mL) and as the highest concentration in a single sample (6.18 × 10^6^ cell/mL). River Tresa was also the river with the highest number of microcolonies (1.44 × 10^6^ microcolonies/mL). Aggregates were instead higher in River Bardello (4.93 × 10^4^ aggregates/mL) than in the other rivers (Table S2).

### Gene abundances

**Figure 2.**
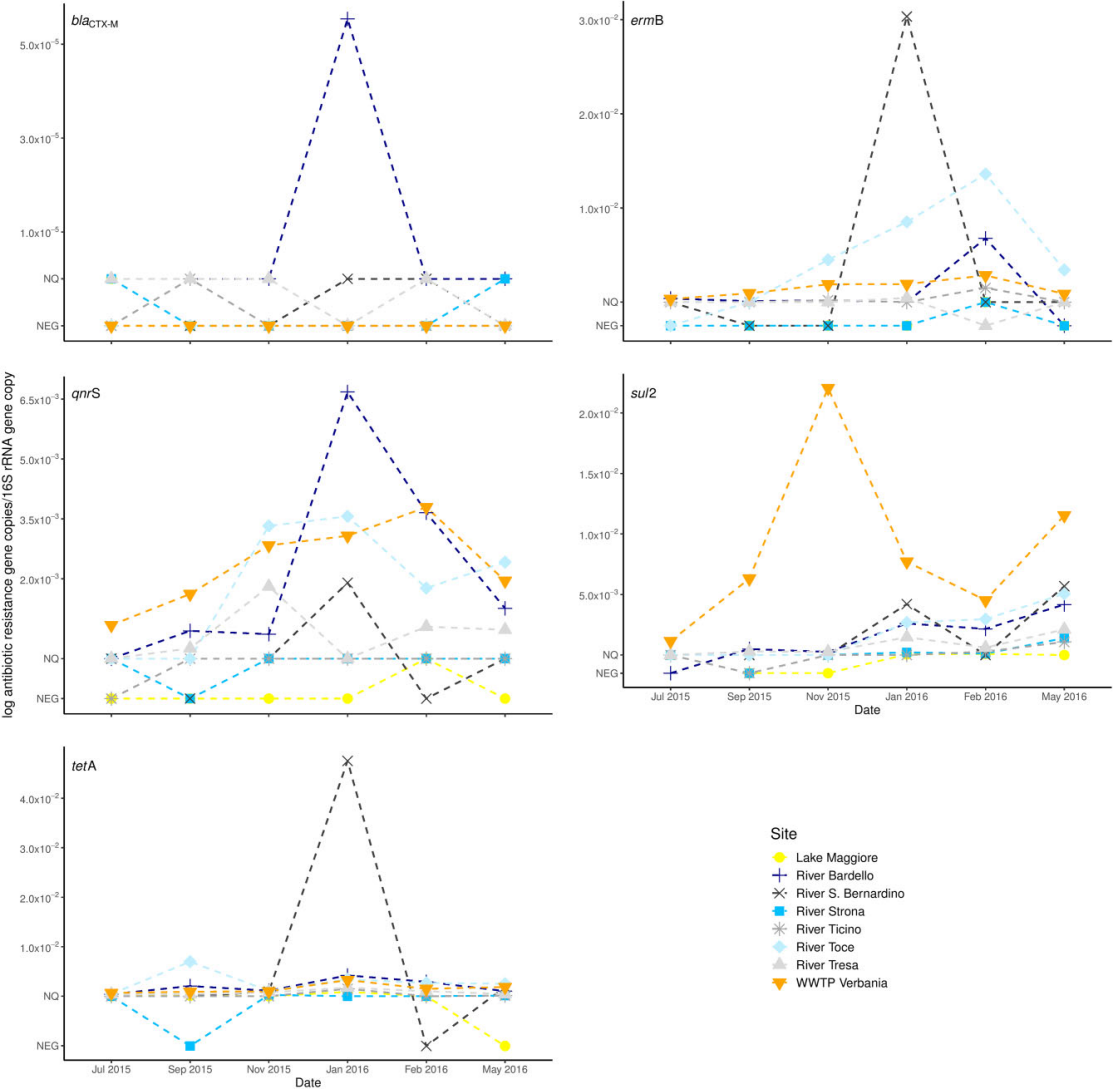
Abundances of the ARGs (*bla*_CTX-M_, *erm*B, *qnr*S, *sul*2, *tet* A). The mean gene abundances (normalized to 16S rRNA gene) measured in different sites and dates. NQ indicates samples that resulted positive but not quantifiable, NEG refer to samples where the gene was not detected.

The *sul*2 and *tet*A genes were present in all the samples from the WWTP effluent and often also in the lake and rivers. When quantifiable, *sul*2 ranged from 5.91×10^−5^ to 2.23×10^−2^ gene copies/16S rRNA gene copy and *tet*A from 1.08×10^−4^ to 4.87×10^−2^ gene copies/16S rRNA gene copy (Fig. 2). The *bla*_CTX-M_ gene was never detected in the samples collected from the WWTP effluent and from River Toce, in the other samples it was only sporadically positive but not quantifiable (it was quantifiable in one sample from River Bardello: 5.54×10^−5^ gene copies/16S rRNA gene copy) (Fig. 2). The *erm*B and *qnr*S genes were present in all the samples from the WWTP effluent and generally positive in the samples collected from the rivers. In Lake Maggiore these genes were, apart for one sample, never detectable. When quantifiable, *erm*B was comprised between 1.04×10^−4^ and 3.08×10^−2^ gene copies/16S rRNA gene copy and *qnr*S between 2.58×10^−4^ and 6.70×10^−3^ gene copies/16S rRNA gene copy (Fig. 2).

### Correlation of the ARG copies/16S rRNA gene copy between Lake Maggiore and the rivers or the effluent of WWTP

Only two genes, *sul*2 and *tet*A, were abundant enough to allow testing the correlation of their relative abundances between the data measured in Lake Maggiore and those in the rivers and in the effluents of the WWTP. The abundance of the *sul*2 gene in Lake Maggiore was not correlated with the one measured in the other sampled sites. By contrast, *tet*A measured in Lake Maggiore was correlated with the one measured in the rivers Bardello, San Bernardino, Ticino and Tresa (all Pearson's *r* ≥ 0.8) and with that determined in the effluents of the WWPT (all Pearson's *r* ≥ 0.87) (Table 1).

**Table 1.** Pearson's *r* correlation between the abundance of resistance genes (*sul*2 and *tet*A) measured in Lake Maggiore and in the rivers and WWTP effluent.

	Correlation Lake Maggiore	
Gene	Site	*r*
*sul*2	Bardello	0.33
	Strona	−0.18
	S. Bernardino	0
	Ticino	−0.10
	Toce	0.38
	Tresa	0.14
	WWTP Verbania	−0.32
*tet*A	Bardello	0.80
	Strona	−0.33
	S. Bernardino	0.99
	Ticino	0.99
	Toce	0.23
	Tresa	0.85
	WWTP Verbania	0.87

### Relationship between biotic and abiotic factors and ARGs

**Figure 3.**
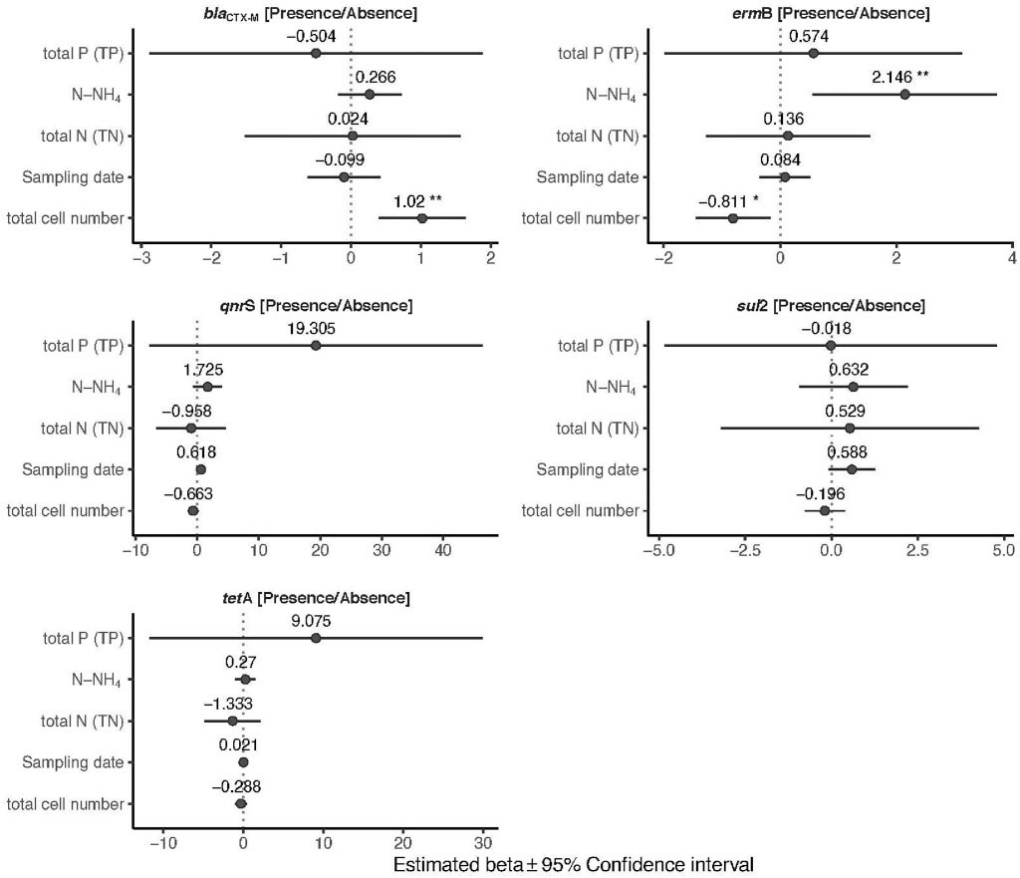
Influence of abiotic and biotic factors on the probability of presence of the target ARGs. Forest plots summarize the estimated parameters based on Bernoulli generalized linear mixed models. Error bars mark confidence intervals. Asterisks mark significant effects (* *P* < 0.05; ** *P* < 0.01). Estimated regression parameters and *P* values are in Supplementary Table 2.

The detection frequency of the *erm*B gene increased significantly with increasing values of N-NH_4_ (*P* = 0.008) and decreased with increasing concentration of bacterial cell (*P* = 0.013) (Fig. 3; Table S3). The detection frequency of the *bla*_CTX-M_ gene rose as the bacterial cell concentration increased (*P* = 0.001) (Fig. 3; Table S3). For *sul*2, *tet*A and *qnr*S genes, no significant effect of the tested biotic and abiotic factors on their presence was observed (Fig. 3; Table S3).

**Figure 4.**
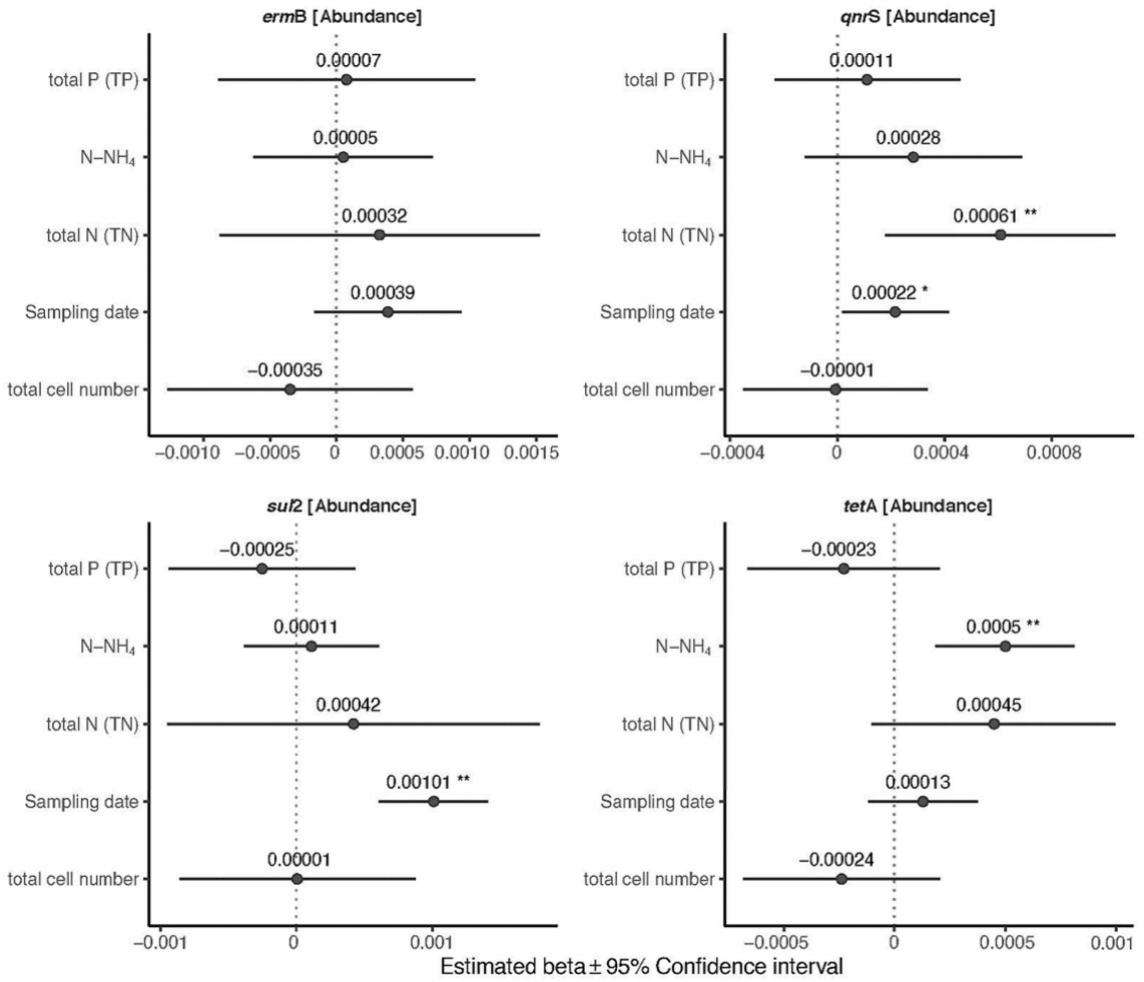
Influence of abiotic and biotic factors on the relative abundance of the target ARGs (normalized to 16SrRNA gene). Forest plots summarize the estimated parameters based on linear mixed models. Error bars mark confidence intervals. Asterisks mark significant effects (* *P* < 0.05; ** *P* < 0.01). Estimated regression parameters and *P* values are in Supplementary Table 3.

The abundances of the *sul*2 and *qnr*S genes were positively associated with sampling date (*P* < 0.001, *P* = 0.033, respectively) (Fig. 4) (Table S4). The *qnr*S gene was also positively influenced by TN (*P* = 0.005) (Fig. 4) (Table S4). The abundance of the *tet*A gene was positively affected by N-NH_4_ (*P* = 0.002) (Fig. 4; Table S4). The abundance of *erm*B was not affected by any of the tested abiotic and biotic factors (Fig. 4; Table S4).

### Influence of catchment-level predictors on ARGs

**Figure 5.**
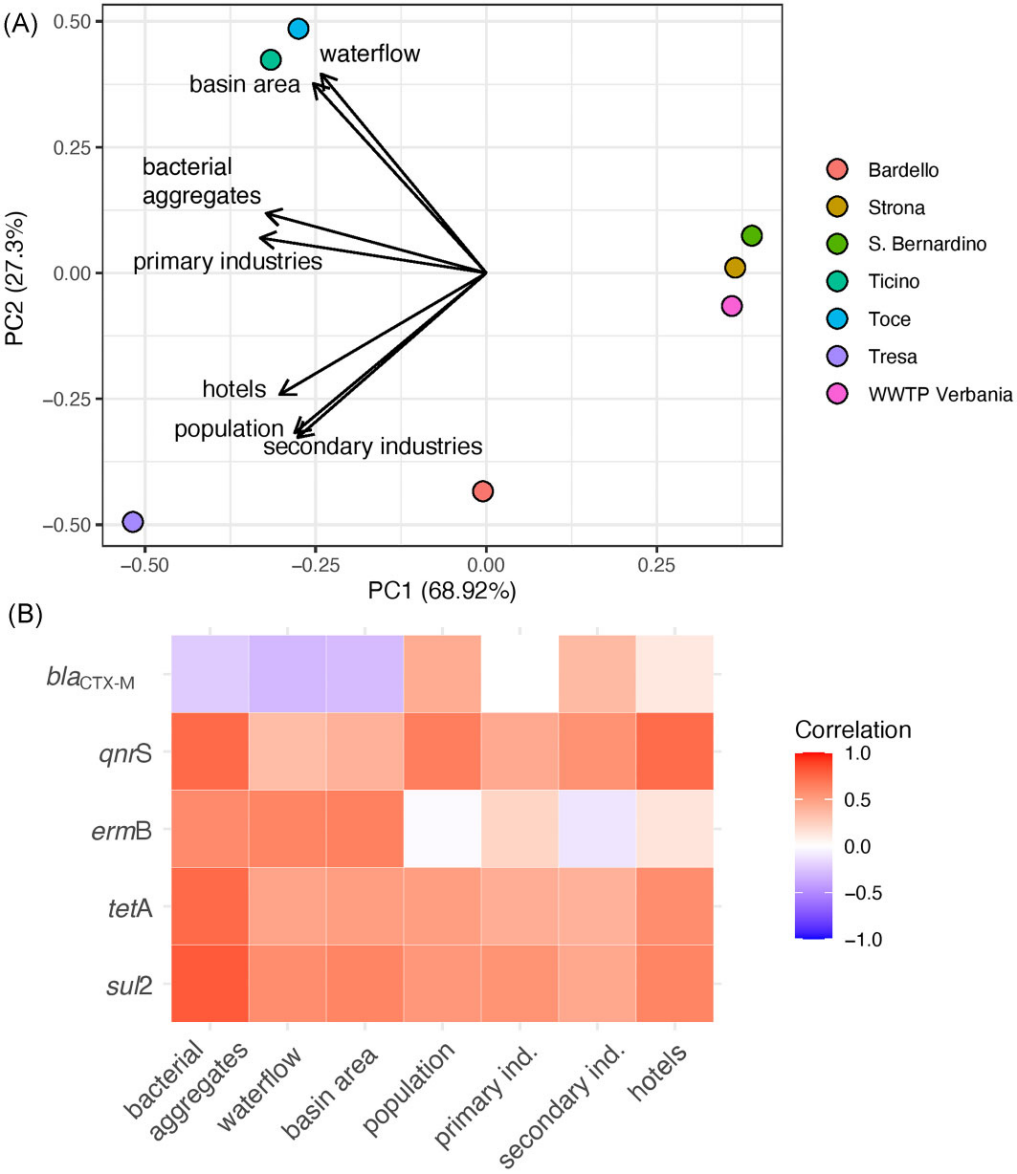
Influence of catchment-level predictors on the abundance of ARGs. (A) Bi-plot of principal component analysis (PCA) scores for the first two axes based on the seven catchment-level variables. The position of each site is marked with colored dots. (B) Heat-map showing Pearson's *r* correlations between the abundance of the five target genes and the seven catchment-level predictors.

The abundances of ARGs in the Lake Maggiore and its tributaries correlated with land use and anthropogenic pressures (Fig. 1, Fig. 5A): while primary industries were not a major driver to define the different watersheds, secondary industries, resident population and touristic activities were impacting more in the watershed of the rivers Tresa and Bardello. Interestingly, while overall numbers and density rather correlated for population and secondary industries across the different river watersheds (high number and density for Bardello and Tresa, low values for Toce and Ticino), the parameters for primary industries and touristic pressure (number of hotels and density) were not proportional among the watersheds. The rivers Tresa and Bardello were also the ones with the highest load of ARGs per cubic meter and, together with the River Toce, with the highest overall values per year. All the ARGs strongly correlated with these parameters (Fig. 5B), while only *erm*B was correlated to the overall presence of primary industries. As a result, the overall ARG load from each watershed (Fig. 1F) was concomitantly influenced by both, overall abundance and density of each pressure predictor, in a modulated way (Fig. 1A–C and E).

**Figure 6.**
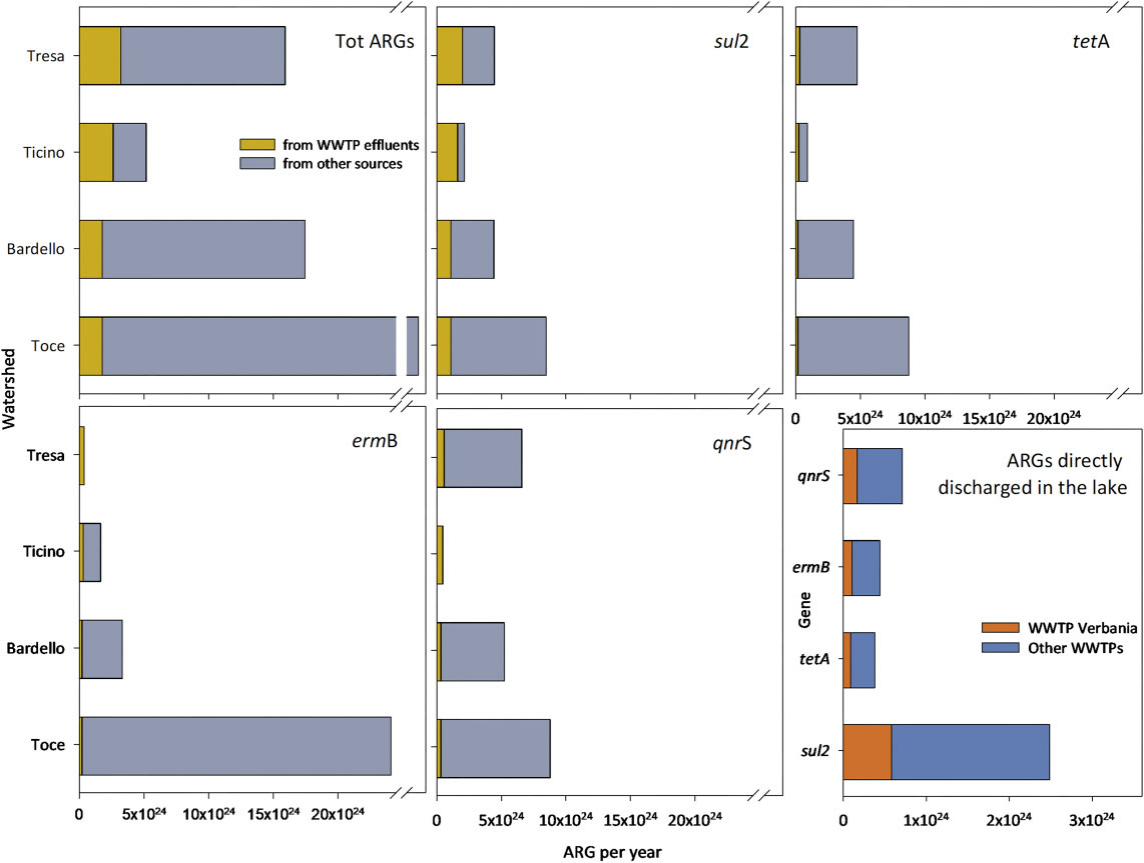
Proportion of ARGs originated directly from WWTP effluents or from other sources. The last panel refers to the number of ARGs released by the WWTP of Verbania, compared with those by other WWTPs directly discharging in Lake Maggiore. The x-axis indicates the extrapolated value of ARGs per year in each sampling site.

Using the effluent of the WWTP of Verbania as proxy of load of ARGs in the effluents of the other WWTPs in the area (the different WWTPs are receiving very comparable inflows, the level of treatment, although some differences, is comparable, as well as the limits they should meet according to the Italian and the Swiss laws on wastewater effluent quality) we defined the ARG load from each large and medium-sized WWTP (considering the water flow of each single WWTP), and thus, within the overall load measured in each watershed (Fig. 1D), we extrapolated the proportion of measured ARGs potentially originated from WWTP effluents. These values are generally limited (<10% of the overall load per year for each considered ARG; Fig. 6) in comparison with the ARGs from other sources of pollution. The proportion of the *sul*2 gene originated from WWTP effluents is higher in all watersheds, but especially in River Ticino (75%), Tresa (44%) and Bardello (25%). A strong prevalence of WWTP effluent-originated genes was detected also for *erm*B in the River Tresa, and for *qnr*S in the River Ticino. The ARGs directly discharged from WWTP effluents in Lake Maggiore include those from the Verbania WWTP (and a few smaller WWTPs) and represent a limited amount when compared with those discharged through the rivers (Fig. 6). Large and medium-sized WWTPs in the watershed of Lake Maggiore and their water flows are listed in Table S5.

## Discussion

The dynamics of the ARGs measured in Lake Maggiore are available from 2013 (detected monthly) and are characterized by large differences among genes (Di Cesare et al. 2015, Eckert et al. 2019a, CIPAIS 2021): bacteria carrying *sul*2 and *tet*A are constantly present and abundant in the waters of the lake in the last 10 years, *bla*_CTX-M_ in 2015–6 was only sporadically quantifiable, missing the seasonality characterizing other years (present in winter/spring, absent for the rest of the year; Eckert et al. 2019). The *sul*2 gene, widely distributed and abundant in the microbiome of the lake, was also discharged in large numbers from the rivers (mainly from Toce, Tresa and Bardello), but without any correlation between loads and abundances in the lake. The occurrence and abundance of *sul*2 in the lake suggest its constitutive presence in the lacustrine bacterial community (where this gene is always detectable, and it can sporadically equal the abundances of the 16SrRNA gene; Di Cesare et al. 2015) and, thus, a general independence of its dynamics from external loads. The other tested genes resulted as generally absent (or non-quantifiable) in the lake, despite the important load we could detect from the watershed and the evidence of their wide distribution in freshwater ecosystems (Proia et al. 2016, Di Cesare et al. 2017, Wang et al. 2020). This shows that the microbial community of the lake is in some way resistant (or resilient) to the stabilization of bacteria carrying these genes, as demonstrated also by the bacterial community of Lake Maggiore at the estuary of River Bardello, which is in fact not affected by the discharge of ARG-polluted waters from the river (Corno et al. 2023). In our article (Corno et al. 2023) we could demonstrate a clear filtering of most ARGs of anthropogenic origin identified in River Bardello, once its waters were entering Lake Maggiore. This could be related to the limited water flow of the river, in comparison with the huge water mass of the lake (dilution effect), but also by the high selective pressure operated by the well-established microbial communities of the lake against the proliferation of allochthonous bacteria (and genes) from the river.

In contrast to *sul*2 and the other measured ARGs, a strong effect on the abundances of the *tet*A gene in the lacustrine bacterial community and in the rivers (and of the effluent of the WWTP) was detected. In fact, *tet*A (and also other ARGs) was positively affected by ammonium (N-NH_4_), a chemical compound generally present in low quantities in pristine waters (Marañón et al. 2006) and showing higher concentrations in the presence of anthropogenic pollution (e.g. wastewaters, leachates, runoff from waste disposal sites and agricultural fields, atmospheric deposition; summarized in Huang et al. 2018). The positive correlation between the presence/abundance of several ARGs and the concentration of N-NH_4_ supports the hypothesis that the anthropogenic contamination of water is one of the main factors explaining the spread of ARG-carrying bacteria. Other factors influencing ARG dynamics were sampling date (suggesting a potential seasonality), in addition to overall bacterial abundance and TN concentrations, both parameters that, in subalpine watersheds, can be related to anthropogenic impact.

To elaborate further, we observed that areas characterized by greater population, higher population density, industrial activity (such as secondary industries) and hotels had released high abundances of most ARGs into the lake. Conversely, areas with high numbers of primary activities like agriculture and mining discharged larger amounts of the *erm*B gene into the lake. This finding suggests that the land use can contribute to the dissemination of diverse antibiotic resistances into the environment, even in areas like the basin of Lake Maggiore, where human activities overlap extensively among the different tributary watersheds.

The (very) low concentrations of heavy metals in the water of Lake Maggiore (CIPAIS 2016, CIPAIS 2017) and of the tributaries, as well as the extremely low concentrations of antibiotics and other pharmaceuticals [overall concentration ~17 ng L^−1^ in Lake Maggiore; S. Castiglioni (personal communication)] suggest a limited direct selective (or co-selective) pressure towards the spread of resistances, and possibly implies their allochthonous origin.

The different river catchments were not only exposed to a certain degree of different human activities but received the effluents of a number of large and medium-sized WWTPs (>10 000 PE), which are generally considered hotspots for the spread of ARGs into the environment (Rizzo et al. 2013). By using the measurements of the ARGs within the bacterial community of the WWTP effluent from Verbania as a model for the effluents in the area, adjusted on the respective water-flow rate of each WWTP effluent, we could approximate the impact of WWTP effluents on the overall ARG load in the bacterial communities of the different rivers. This was possible because, according to the data available on the effluents of other WWTPs in the area (Gravellona Toce, Cannobio, Bioggio, Giubiasco, Gavirate), we had evidence of a comparable load and distribution of ARGs in the different effluents (qPCR and shotgun metagenomic data from Corno et al. 2019, 2023, CIPAIS 2021). Surprisingly, with the exclusion of a few cases and, generally, of the *sul*2 gene, the contribution of the WWTP effluents to the overall ARG numbers was extremely limited. This is in contrast to the generally accepted assumption that presents WWTP effluents among the most prominent sources of ARGs of anthropogenic origin into the environment (Karkman et al. 2018). The overall impact in terms of spread of ARG within the microbiome of the lake caused by WWTP effluents that discharge directly into the lake was also limited. This does not mean that WWTP effluents are not a hotspot for the selection of ARGs in potentially pathogenic bacteria, which has been well demonstrated to date (Proia et al. 2018, Bengtsson-Palme et al. 2019, Alexander et al. 2020), but rather that their impact as a source of antibiotic resistances in high-income countries with efficient WWTPs is limited both quantitatively and (probably) spatially, compared with other sources, whether they are diffuse, point sources, or occasional.

It is indeed possible that, especially in disturbed ecosystems (e.g. River Bardello), an immediate increment of ARGs from the WWTP effluents is promoted by the sudden growth of antibiotic-resistant bacteria released with the treated wastewater (Di Cesare et al. 2023), but this situation is unlikely to happen in more complex and stable environments like River Toce and River Ticino, and even more so River Tresa, where the anthropogenic impact from the upper part of the watershed is buffered by the presence of a deep large lake (Lake Lugano) and its structured and ecologically resistant microbial community (Corno et al. 2019). Still, even for River Bardello, the estimated contribution in terms of ARGs by the two WWTP effluents directly discharging into the river to the overall ARGs number measured at the river's mouth in the lake is limited.

Our findings lend support to the findings presented by Lee et al. (2022) in Swiss rivers, which indicated that the impact of untreated sewage from wastewater bypasses on the levels of ARGs in the environment can be greater over the course of a year than the impact caused by treated effluents. Given that conventional wastewater treatments can reduce ARGs by 80–99% (Di Cesare et al. 2016b, Sabri et al. 2020), it is plausible that heavy rainfall and subsequent activation of water bypasses lasting 10–20 days in a year can cause a discharge into the environment of a similar amount of ARGs as the effluents of their otherwise receiving WWTPs.

Additionally, our results highlight the direct contribution of water runoff from anthropogenic activities such as agriculture, industry and roads to the presence of ARGs in the rivers. This underscores the need for further research to quantify and identify the specific types of ARGs released by these activities, looking for a possible correlation between the fate of allochthonous resistant bacteria in lakes and rivers and the magnitude of water pollution, of anthropogenic pressures and, in general, to the ecological instability of any environment exposed to high levels of stress.

Ultimately, our findings suggest that the current use and design of water bypasses must be re-evaluated, and that WWTP effluents should no longer be considered as the primary, and sometimes the only, source of ARG release into surface waters.

## Supplementary Material

fiae025_Supplemental_Files
