# Supplementary material for: Where do the antibiotic resistance genes come from? A modulated analysis of sources and loads of resistances in Lake Maggiore
Source: FEMS Microbiol Ecol. 2024 Feb 22;100(4):fiae025. doi: 10.1093/femsec/fiae025 (PMC10939355; doi:10.1093/femsec/fiae025)
Supplement: fiae025_Supplemental_Files [file fiae025_supplemental_files.zip › Supplementary_data Material2.docx]

**Supplementary Material**

**Where do the antibiotic resistance genes come from? A modulated analysis of sources and loads of resistances in Lake Maggiore**

Andrea Di Cesare ^a*^, Stefano Mammola ^a,b^, Raffaella Sabatino ^a^, Diego Fontaneto ^a^, Ester M. Eckert ^a^, Michela Rogora ^a^, Tiziana Tonsi ^a^, Gianluca Corno ^a*^

^a^ National Research Council of Italy – Water Research Institute (CNR-IRSA), Verbania, Italy

^b^ Finnish Museum of Natural History (LUOMUS), University of Helsinki, Helsinki, Finland

* Corresponding author. CNR – Water Research Institute, Largo Tonolli 50, 28922, Verbania, Italy. Email address: gianluca.corno@cnr.it


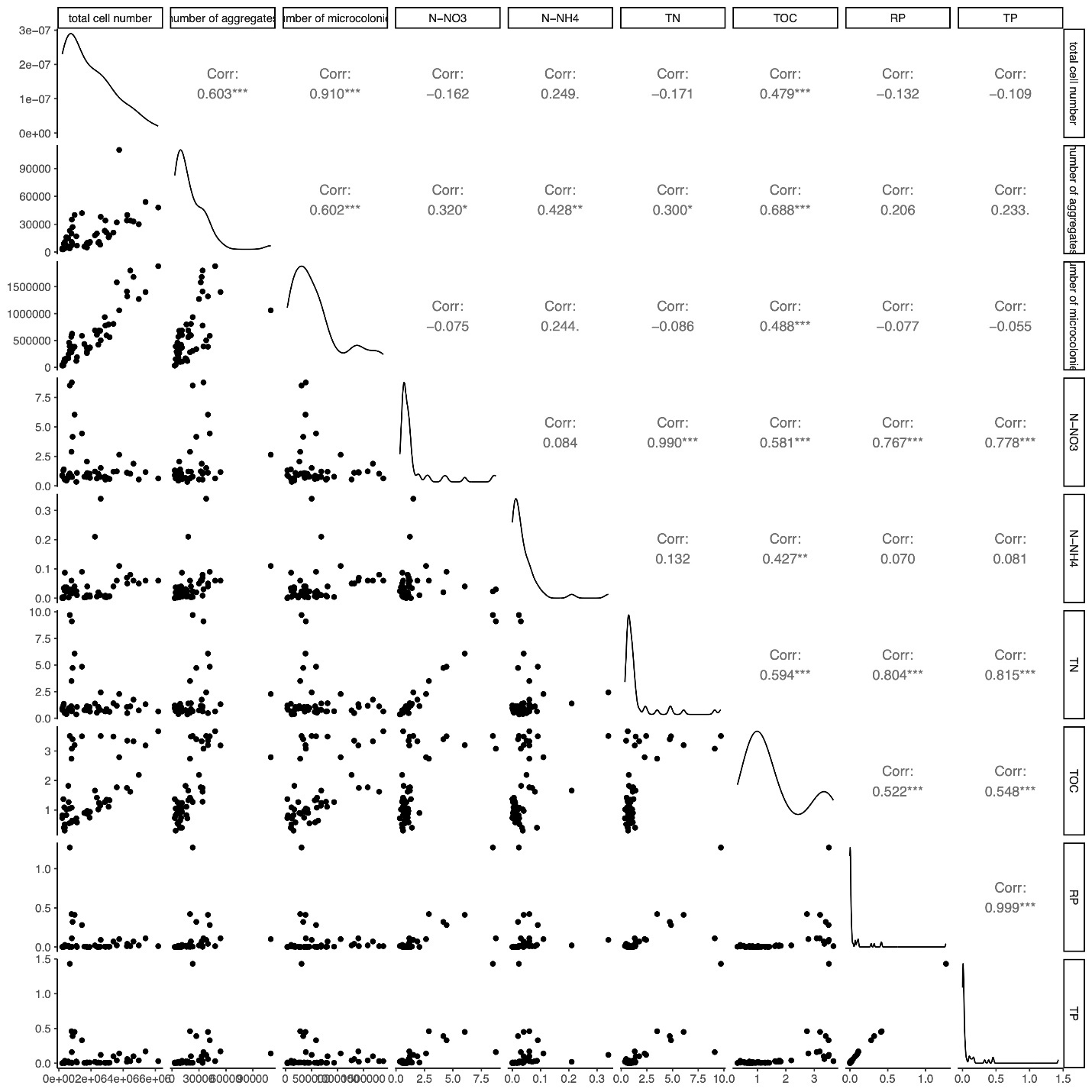


**Figure S1.** Pairwise comparisons between the variables included in the analyses. Numbers in the squares indicate Pearson’s coefficient between a pair of variables.

**Table S3.** Estimated regression parameters for the Bernoulli generalized linear mixed models modeling the relationship between the presence/absence of the target ARGs and the abiotic and biotic factors. SE = standard error; CI = 95% confidence interval; p = p.value.

| **Model** | **Parameter** | **Beta** | **SE** | **CI_low** | **CI_high** | **z** | **p** |
| --- | --- | --- | --- | --- | --- | --- | --- |
| *sul*2 [Presence/Absence] | Intercept | 1.281 | 0.491 | 0.317 | 2.244 | 2.606 | 0.009 |
| *sul*2 [Presence/Absence] | Sampling date | 0.588 | 0.339 | -0.077 | 1.252 | 1.734 | 0.083 |
| *sul*2 [Presence/Absence] | total cell number | -0.196 | 0.293 | -0.77 | 0.378 | -0.669 | 0.503 |
| *sul*2 [Presence/Absence] | total N (TN) | 0.529 | 1.907 | -3.209 | 4.266 | 0.277 | 0.782 |
| *sul*2 [Presence/Absence] | N-NH_4_ | 0.632 | 0.803 | -0.942 | 2.206 | 0.787 | 0.431 |
| *sul*2 [Presence/Absence] | total P (TP) | -0.018 | 2.458 | -4.836 | 4.799 | -0.008 | 0.994 |
| *tet*A [Presence/Absence] | Intercept | 3.51 | 3.011 | -2.391 | 9.411 | 1.166 | 0.244 |
| *tet*A [Presence/Absence] | Sampling date | 0.021 | 0.229 | -0.429 | 0.47 | 0.09 | 0.928 |
| *tet*A [Presence/Absence] | total cell number | -0.288 | 0.366 | -1.005 | 0.429 | -0.788 | 0.431 |
| *tet*A [Presence/Absence] | total N (TN) | -1.333 | 1.789 | -4.839 | 2.172 | -0.746 | 0.456 |
| *tet*A [Presence/Absence] | N-NH_4_ | 0.27 | 0.626 | -0.958 | 1.497 | 0.431 | 0.666 |
| *tet*A [Presence/Absence] | total P (TP) | 9.075 | 10.603 | -11.705 | 29.856 | 0.856 | 0.392 |
| *erm*B [Presence/Absence] | Intercept | 0.632 | 0.359 | -0.072 | 1.335 | 1.759 | 0.079 |
| *erm*B [Presence/Absence] | Sampling date | 0.084 | 0.223 | -0.353 | 0.52 | 0.376 | 0.707 |
| *erm*B [Presence/Absence] | total cell number | -0.811 | 0.325 | -1.448 | -0.174 | -2.497 | 0.013 |
| *erm*B [Presence/Absence] | total N (TN) | 0.136 | 0.719 | -1.274 | 1.546 | 0.189 | 0.85 |
| *erm*B [Presence/Absence] | N-NH_4_ | 2.146 | 0.808 | 0.563 | 3.729 | 2.657 | 0.008 |
| *erm*B [Presence/Absence] | total P (TP) | 0.574 | 1.306 | -1.986 | 3.134 | 0.44 | 0.66 |
| *qnr*S [Presence/Absence] | Intercept | 7.071 | 4.422 | -1.597 | 15.738 | 1.599 | 0.11 |
| *qnr*S [Presence/Absence] | Sampling date | 0.618 | 0.323 | -0.015 | 1.251 | 1.915 | 0.056 |
| *qnr*S [Presence/Absence] | total cell number | -0.663 | 0.451 | -1.547 | 0.221 | -1.469 | 0.142 |
| *qnr*S [Presence/Absence] | total N (TN) | -0.958 | 2.886 | -6.615 | 4.698 | -0.332 | 0.74 |
| *qnr*S [Presence/Absence] | N-NH_4_ | 1.725 | 1.197 | -0.622 | 4.071 | 1.44 | 0.15 |
| *qnr*S [Presence/Absence] | total P (TP) | 19.305 | 13.788 | -7.719 | 46.329 | 1.4 | 0.161 |
| *bla*_CTXM_ [Presence/Absence] | Intercept | -1.354 | 0.377 | -2.093 | -0.615 | -3.593 | <0.001 |
| *bla*_CTXM_ [Presence/Absence] | Sampling date | -0.099 | 0.265 | -0.618 | 0.42 | -0.375 | 0.708 |
| *bla*_CTXM_ [Presence/Absence] | total cell number | 1.02 | 0.317 | 0.399 | 1.642 | 3.22 | 0.001 |
| *bla*_CTXM_ [Presence/Absence] | total N (TN) | 0.024 | 0.788 | -1.521 | 1.569 | 0.03 | 0.976 |
| *bla*_CTXM_ [Presence/Absence] | N-NH_4_ | 0.266 | 0.232 | -0.188 | 0.721 | 1.149 | 0.25 |
| *bla*_CTXM_ [Presence/Absence] | total P (TP) | -0.504 | 1.214 | -2.884 | 1.876 | -0.415 | 0.678 |

**Table S4.** Estimated regression parameters for the linear mixed models modeling the relationship between the abundance of the target ARGs and the abiotic and biotic factors. SE = standard error; CI = 95% confidence interval; p = p.value.

| **Model** | **Parameter** | **Beta** | **SE** | **CI_low** | **CI_high** | **z** | **p** |
| --- | --- | --- | --- | --- | --- | --- | --- |
| *sul*2 [Abundance] | Intercept | 0.001543 | 0.000593 | 0.000381 | 0.002704 | 2.602969 | 0.009242 |
| *sul*2 [Abundance] | Sampling date | 0.00101 | 0.000205 | 0.000608 | 0.001412 | 4.929407 | 0.000001 |
| *sul*2 [Abundance] | total cell number | 0.000007 | 0.000443 | -0.000862 | 0.000876 | 0.015415 | 0.987701 |
| *sul*2 [Abundance] | total N (TN) | 0.00042 | 0.000698 | -0.000948 | 0.001788 | 0.602 | 0.547174 |
| *sul*2 [Abundance] | N-NH_4_ | 0.00011 | 0.000254 | -0.000388 | 0.000609 | 0.433011 | 0.665006 |
| *sul*2 [Abundance] | total P (TP) | -0.000253 | 0.00035 | -0.00094 | 0.000434 | -0.721649 | 0.47051 |
| *tet*A [Abundance] | Intercept | 0.000972 | 0.000281 | 0.00042 | 0.001524 | 3.453252 | 0.000554 |
| *tet*A [Abundance] | Sampling date | 0.000128 | 0.000127 | -0.000121 | 0.000378 | 1.006612 | 0.314121 |
| *tet*A [Abundance] | total cell number | -0.00024 | 0.000226 | -0.000683 | 0.000204 | -1.060011 | 0.289139 |
| *tet*A [Abundance] | total N (TN) | 0.000448 | 0.00028 | -0.000102 | 0.000997 | 1.597467 | 0.110162 |
| *tet*A [Abundance] | N-NH_4_ | 0.000499 | 0.00016 | 0.000186 | 0.000812 | 3.125384 | 0.001776 |
| *tet*A [Abundance] | total P (TP) | -0.000229 | 0.000222 | -0.000665 | 0.000206 | -1.033239 | 0.301492 |
| *erm*B [Abundance] | Intercept | 0.000986 | 0.000549 | -0.000091 | 0.002062 | 1.794775 | 0.072689 |
| *erm*B [Abundance] | Sampling date | 0.000386 | 0.000282 | -0.000166 | 0.000938 | 1.369695 | 0.170782 |
| *erm*B [Abundance] | total cell number | -0.000348 | 0.00047 | -0.001269 | 0.000573 | -0.740155 | 0.459206 |
| *erm*B [Abundance] | total N (TN) | 0.000323 | 0.000613 | -0.000878 | 0.001524 | 0.526682 | 0.598415 |
| *erm*B [Abundance] | N-NH_4_ | 0.000051 | 0.000344 | -0.000623 | 0.000725 | 0.148376 | 0.882046 |
| *erm*B [Abundance] | total P (TP) | 0.000075 | 0.000492 | -0.000889 | 0.00104 | 0.152619 | 0.878699 |
| *qnr*S [Abundance] | Intercept | 0.000779 | 0.000206 | 0.000376 | 0.001182 | 3.786196 | 0.000153 |
| *qnr*S [Abundance] | Sampling date | 0.000216 | 0.000101 | 0.000017 | 0.000415 | 2.129692 | 0.033197 |
| *qnr*S [Abundance] | total cell number | -0.000007 | 0.000176 | -0.000351 | 0.000337 | -0.041366 | 0.967004 |
| *qnr*S [Abundance] | total N (TN) | 0.000608 | 0.000219 | 0.000178 | 0.001037 | 2.772176 | 0.005568 |
| *qnr*S [Abundance] | N-NH_4_ | 0.000284 | 0.000206 | -0.000121 | 0.000688 | 1.375801 | 0.168883 |
| *qnr*S [Abundance] | total P (TP) | 0.000111 | 0.000177 | -0.000235 | 0.000458 | 0.629453 | 0.529053 |

**Table S5.** Medium-large size WWTPs in Lake Maggiore basin. Data on their size (in population equivalent, used to determine the dimension) and on the flow rate of their effluents from <https://www4.ti.ch/dt/da/spaas/upaai/temi/acqua-protezione-e-approvvigionamento/per-saperne-di-piu/documentazione/impianti-di-depurazione-ida>, from <https://www.alfavarese.it/wp-content/uploads/2022/10/Schede-impianti-di-depurazione-ALFA2.pdf> and from <https://www.acquanovaravco.eu/Pagina/depurazione>. The last two WWTPs in the table (in red) are in watersheds not considered for this study.

| Medium-large WWTPs | | | |
| --- | --- | --- | --- |
| Location | **Water basin receiving the effluents** | **Waterflow m^3^ s^-1^** | **Waterflow m^3^ year^-1^** |
| Domodossola | Toce | 0.21 | 6529193 |
| Gravellona | Toce | 0.18 | 5719573 |
| Omegna | Toce | 0.26 | 8161491 |
| Villadossola | Toce | 0.11 | 3591056 |
| Gavirate | Bardello | 0.41 | 13058385 |
| Monvalle | Bardello | 0.09 | 2762554 |
| Foce Ticino | Ticino | 0.22 | 6855652 |
| Giubiasco | Ticino | 0.33 | 10446708 |
| Biasca | Ticino | 0.20 | 6170087 |
| Barbengo | Tresa | 0.19 | 5876273 |
| Bioggio | Tresa | 0.51 | 15996522 |
| Luino | Tresa | 0.13 | 4232131 |
| Ferrera | Tresa | 0.09 | 2869776 |
| Verbania | **Lake Maggiore** | **0.27** | **8514720** |
| Locarno | Lake Maggiore | 0.46 | 14364224 |
| Cannobio | Lake Maggiore | 0.20 | 6352904 |
| Dormelletto | Lake Maggiore | 0.23 | 7182112 |
| Laveno | other basins | 0.17 | 5280388 |
| Casalzuigno | other basins | 0.08 | 2459808 |
